# Metal and Metal Oxide Nanoparticles Using *Artemisia* Species: Synthesis, Characterization and Applications

**DOI:** 10.3390/plants15040600

**Published:** 2026-02-13

**Authors:** Delia Luca, Roxana Jijie, Gabriela Vochita, Lucia-Florina Popovici, Marius-Nicusor Grigore, Lacramioara Oprica

**Affiliations:** 1 Doctoral School of Biology, Faculty of Biology, Alexandru Ioan Cuza University of Iasi, Bd. Carol I, 700505 Iasi, Romania; delia.luca@student.uaic.ro (D.L.);; 2 Research Center on Advanced Materials and Technologies (RAMTECH), Department of Exact and Natural Sciences, Institute of Interdisciplinary Research, Alexandru Ioan Cuza University of Iasi, Bd. Carol I, 700506 Iasi, Romania; roxanajijie@yahoo.com; 3 Institute of Biological Research Iasi, Branch of NIRDBS Bucharest, 47 Lascar Catargi, 700107 Iasi, Romania; 4 Department of Agricultural Sciences & Food Engineering, Lucian Blaga University of Sibiu, 72 Ion Ratiu, 550024 Sibiu, Romania; luciaflorina.popovici@ulbsibiu.ro; 5 Department of Biology, Faculty of Biology, Alexandru Ioan Cuza University of Iasi, Bd. Carol I, 700505 Iasi, Romania

**Keywords:** biogenic nanoparticles, genus *Artemisia*, biosynthesis method, applications

## Abstract

This review aims to explore the green synthesis of metal and metal oxide nanoparticles using various species of the genus *Artemisia*. The synthesis processes commonly involve aqueous or organic extracts of plant parts (e.g., leaves, stems, and roots), which react with metal salt solutions (e.g., AgNO_3_, Zn(NO_3_)_2_, HAuCl_4_, Cu(NO_3_)_2_) under controlled parameters, including pH, temperature, and light exposure. The synthesized nanoparticles are characterized using techniques such as UV–Visible spectroscopy, transmission electron microscopy (TEM), scanning electron microscopy (SEM), Fourier transform infrared spectroscopy (FTIR), X-ray diffraction (XRD), dynamic light scattering (DLS), and zeta potential analysis. These approaches provide information on nanoparticle size, morphology, crystallinity, surface chemistry and charge, which are significantly influenced by synthesis parameters and the specific *Artemisia* species used. The biosynthesized nanoparticles have demonstrated promising multifunctional applications, including broad-spectrum antimicrobial activity against bacterial and fungal strains, antioxidant capacity, anticancer potential, as well as applications in agriculture and environmental remediation.

## 1. Introduction

Different mitigation approaches are employed to enhance global agricultural crop productivity and assist environmental remediation [1]. Nanotechnology encompasses the design, synthesis, and manipulation of materials at the nanometric scale for the fabrication, characterization, and application of a wide range of nanoparticles (NPs). In recent years, metal nanoparticles have attracted considerable interest due to their unique physicochemical properties dependent on their small size, shape, and specific surface area [2,3]. While achieving high monodispersity is a critical goal for diverse applications (e.g., targeted drug delivery, electronics), the uniformity of particles depends significantly on the synthesis method [4]. Physical methods, such as laser ablation, offer the advantage of high purity but often require high energy consumption and can result in broad size distributions (polydispersity) depending on the experimental conditions [5]. In contrast, chemical fabrication methods generally provide superior control over nucleation and growth steps, allowing improved monodispersity and higher yields compared with physical techniques. However, conventional chemical routes often involve hazardous reducing agents and stabilizers, raising environmental and safety concerns [6].

Over the past decade, the principles of green chemistry have been intensively explored in the context of sustainable development [7]. Consequently, increasing efforts have been directed toward the production of non-toxic NPs using environmentally friendly biological approaches. The biosynthesis of nanoparticles employing plant extracts represents a viable alternative to conventional chemical and physical methods. This approach typically requires the use of an eco-friendly solvent (most commonly water), a non-toxic reducing agent, and a suitable stabilizing agent [8]. Beyond the environmental advantages, plant-mediated synthesis offers significant economic benefits. Many plant sources are abundant, renewable, and can be harvested at a low cost, providing a “waste-to-wealth” pathway for large-scale NP production. Moreover, the presence of diverse primary and secondary metabolites in plant extracts can contribute to improved NP stability and may reduce agglomeration and toxicity [9].

Accordingly, the field of nanotechnology is undergoing rapid advancement, and there is increasing attention toward the development of green synthesis approaches for producing metal and metal oxide nanoparticles using plant-based resources. The genus *Artemisia* is taxonomically classified within the family *Asteraceae* (*Compositae*), including over 500 species [10]. Members of this genus are predominantly adapted to temperate climates and are widely distributed across Europe, East Asia, North America, North Africa, and Australia. The highest species diversity is observed in Asia, particularly in China, where approximately 150 species have been recorded, while Europe hosts about 57 species [10,11]. Based on global distribution data, the majority of *Artemisia* species are concentrated in the Northern Hemisphere, whereas only about 25 species are found in the Southern Hemisphere [12].

Plants belonging to the genus *Artemisia* are well known for their richness in bioactive compounds, many of which contribute to their significant medicinal value and diverse biological activities. To date, over 260 *Artemisia* species have been investigated for their essential oil composition and secondary metabolite profiles. Various classes of bioactive compounds have been identified, including terpenoids, flavonoids, coumarins, phenolic acids, alkaloids, essential oils, and sterols (Table A1) [13]. Due to their rich phytochemical content, *Artemisia* species extracts have emerged as promising candidates for the biosynthesis of NPs. These compounds serve as both reducing agents (converting metal ions to NPs) and capping agents (stabilizing the NPs) [14,15].

Research suggests that the *Artemisia* genus is a valuable resource for the synthesis of multifunctional NPs with applications across various sectors. Among the nanoparticles synthesized using *Artemisia* extracts, silver nanoparticles (AgNPs) are the most studied. These NPs have demonstrated effective antimicrobial properties against both Gram-positive and Gram-negative bacterial strains and fungal species [16,17], as well as antioxidant activity [18] and antitumoral properties [19]. Furthermore, they show significant potential in environmental remediation [20], agriculture, and food safety [18,21].

**Figure 1:**
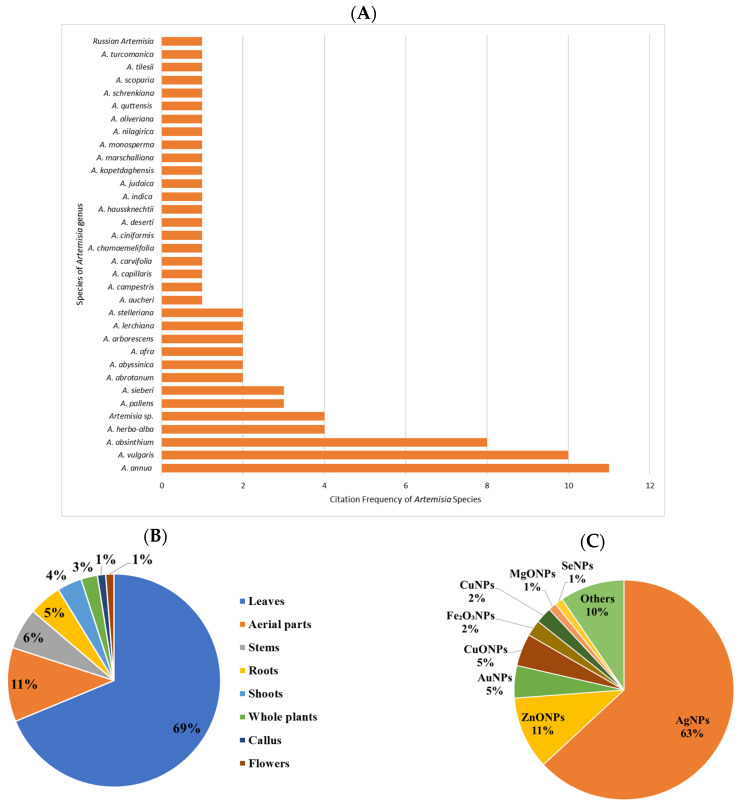
(**A**) The most mentioned *Artemisia* species in terms of number involved in the biosynthesis of NPs. (**B**) Different parts of the plant and (**C**) types of metal/metal oxide-based NPs synthesized using *Artemisia* species extracts.

According to the literature reviewed, among the 34 documented *Artemisia* species used for nanoparticles synthesis, the most common were *A. annua*, *A. vulgaris*, and *A. absinthium (*Figure 1A). Furthermore, while phytochemicals are distributed in different plant organs (e.g., leaves, stems, flowers, fruits and roots), the majority of studies (69%) utilized leaves, followed by aerial parts (11%), for plant-mediated nanoparticles (Figure 1B).

The consulted literature highlights the synthesis and characterization of diverse Artemisia-derived NPs, including AgNPs, silver oxide nanoparticles (Ag_2_ONPs), copper nanoparticles (CuNPs), copper oxide nanoparticles (CuONPs), gold nanoparticles (AuNPs), titanium dioxide nanoparticles (TiO_2_NPs), selenium nanoparticles (SeNPs), zinc oxide nanoparticles (ZnONPs), and tin oxide nanoparticles (TONPs). Of all nanoparticles synthesized via green methods using this genus, most were AgNPs (63%), followed by ZnONPs (11%) and AuNPs (5%) (Figure 1C).

Despite the proliferation of individual studies on the genus Artemisia, a significant knowledge gap exists regarding comparative analyses that evaluate the efficacy of different species and plant parts against various metal precursors. The novelty of this paper lies in addressing this gap through a comprehensive analysis of metal and metal oxide NP synthesis using extracts from various parts of Artemisia species. Additionally, this work details the synthesis methods, physicochemical characteristics (size, shape, zeta potential), and diverse applications, ranging from medical and antimicrobial uses to environmental remediation and food safety.

## 2. Synthesis of Metal and Metal Oxide NPs

**Figure 2:**
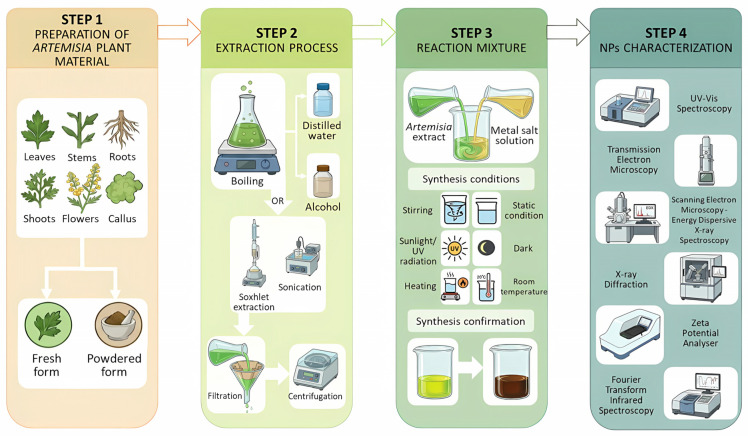
Schematic representation of the workflow for the green synthesis of nanoparticles using *Artemisia* species.

According to the literature, various methods for NP synthesis using extracts from genus *Artemisia* have been documented, usually following four main steps (Figure 2). The initial step involves the collection and preparation of plant material, which may include leaves [22,23,24], stems [25], roots [26,27], aerial parts [28,29], flowers [30], callus [31] or the whole plant [32]. This material can be utilized either fresh [20,33,34] or in powdered form [26,35]. Next, the plant material undergoes an extraction process, mostly through boiling in organic or inorganic solvents (e.g., distilled water [15,36], ethanol [37,38], or methanol [39,40]). Other methods include Soxhlet extraction [41,42] or sonication [30], followed by filtration and centrifugation. The chemical composition of the extract significantly influences the efficiency of NP synthesis. Secondary metabolites, including flavonoids, alkaloids, terpenoids, and phenolic acids, play a crucial role in the reduction and stabilization of metal ions into NPs [39]. Furthermore, specific functional groups, such as amino (-NH_2_), carboxyl (-COOH), carbonyl (-CO), hydroxyl (-OH), and thiol (-SH), enhance the reducing and capping capacity of phytoconstituents, facilitating the formation of stable, non-agglomerated NPs [23]. Metal salt solutions, such as silver nitrate (AgNO_3_), chloroauric acid (HAuCl_4_), copper nitrate (Cu(NO_3_)_2_), zinc acetate (Zn(CH_3_COO)_2_), and iron (III) chloride (FeCl_3_), are prepared at varying concentrations. To induce synthesis, the reaction mixtures are maintained under either continuous stirring [43,44,45] or static conditions while being subjected to specific environmental parameters. These parameters often include exposure to sunlight/UV radiation [31,35,46,47] or dark conditions [48,49], heating [38,50,51] or maintenance at room temperature [44,52]. The formation of NPs is typically indicated by a visible color change in the reaction solution. Depending on the metal and species used, the solution may transform from colorless, yellow, blue, orange, or pale green to final shades of pink, violet, reddish-brown, grey-black, or dark brown. This optical shift is attributed to surface plasmon resonance (SPR), which is directly influenced by the size and shape of the synthesized NPs [14,53,54,55].

Among the phytoconstituent classes present in species of the *Artemisia* genus, terpenoids and flavonoids are notably abundant (Table A1) [56]. These bioactive compounds participate in numerous chemical processes, including the synthesis of NPs. During NP formation, functional groups such as -OH and -CO from flavonoids and other antioxidants donate electrons to metal ions (e.g., Ag^+^, Au^3+^, Cu^2+^, Zn^2+^), reducing them to metallic or metal oxide nanoparticles [15,20,23]. Simultaneously, these phytochemicals adsorb onto NP surfaces, acting as capping and stabilizing agents that prevent aggregation and help control particle size and morphology [57].

Fourier transform infrared spectroscopy (FTIR) has confirmed the interaction of functional groups in metal reduction and stabilization processes through specific absorption bands [14]. As summarized in Table 1, key functional groups include O–H stretching of phenols and alcohols (broad peaks between 3206 and 3472 cm^−1^), C–H stretching of aromatic and methylene groups (2853–3078 cm^−1^; 2072 cm^−1^), and the C=O carbonyl stretching of amide and phenolic compounds (1635–1661 cm^−1^). The spectra also reveal C=C stretching of aromatic rings (~1607 cm^−1^), carboxylate –COO^−^ groups (~1580 cm^−1^), N–O stretching of nitro compounds (1319–1504 cm^−1^), C≡C stretching (2118 cm^−1^), and C–O/C–O–C stretching (1026–1243 cm^−1^; 588 cm^−1^) [14,17,20,27,45]. Furthermore, the successful synthesis of metal and metal oxide nanoparticles is definitively proven by the emergence of characteristic metal–oxygen vibration bands (e.g., Cu–O at 1385 cm^−1^, Ag–O at 506.55 cm^−1^, and Zn–O at 661.84 cm^−1^), confirming that these organic constituents effectively reduce metal ions and stabilize the resulting nanostructures [14,20,45].

The yield of NP synthesis is affected by various factors, including pH, temperature, and the concentration of both plant extract and metallic salt solution. For instance, Avitabile et al. reported that approximately 60% of initial silver was converted into AgNPs after six centrifugation cycles [58]. Similarly, a precursor salt concentration of 0.1 M resulted in the highest production of CuONPs (58%), whereas higher concentrations inhibited synthesis [14]. Regarding reactant ratios, Ali et al. found that an extract to AgNO_3_ ratio of 6:4 (*v*/*v*) achieved the highest conversion efficiency [59]. Furthermore, Kobylinska et al. [26] demonstrated the important role of the solvent. A 70% water–ethanol mixture extracted more bioactive compounds, especially flavonoids, from *A. annua* and *A. tilesii* “hairy” roots culture, thereby increasing the reduction of Ag^+^ and stabilizing the NPs [26]. In terms of pH, no AgNPs were formed at pH levels below 4. However, Ag^+^ reduction increased in alkaline media, with pH 10 being optimal [57].

## 3. *Artemisia*-Based Nanoparticle Characterization

To determine the physicochemical characteristics of synthesized NPs (e.g., size, shape, crystallinity, capping, and zeta potential), a variety of techniques are employed, including UV-Vis spectroscopy, scanning electron microscopy (SEM) with energy-dispersive X-ray spectroscopy (SEM-EDX), transmission electron microscopy (TEM), Fourier transform infrared spectroscopy (FTIR), X-ray diffraction (XRD), dynamic light scattering (DLS), among others.

### 3.1. Size

TEM analysis revealed that the smallest AgNPs (0.60 nm) were obtained from *A. schrenkiana* extract [24], while the largest AgNPs (~200 nm) formed using *A. vulgaris* extract [60]. Furthermore, MgONPs, synthesized with *A. abrotanum* extract, did not exceed 10 nm [46]. The smallest ZnONPs measured 17 nm [39], while Shelembe et al. [55] obtained ZnONPs and Fe_2_O_3_NPs recording 31 nm. Using SEM analysis, CuONPs synthesized from *A. haussknechtii* extract measured approximately 35 nm [49]. By contrast, SeNPs and TiO_2_NPs exhibited larger sizes, 110 nm and 92.58 ± 56.98 nm, respectively [42,49].

Various factors influence particle size. Higher extract concentrations tend to produce larger nanoparticles [24]. Conversely, increasing the volume of *Artemisia* extract relative to the metal precursor often reduces NP size [15]. Regarding pH, alkaline conditions (pH 7–9) favor the synthesis of smaller NPs compared to acidic conditions [50]. Temperature also plays a key role; higher temperatures generally favor smaller particles [50]. However, excessive temperatures may degrade the phytochemicals, thereby compromising the quality and stability of the synthesized NPs, whereas lower temperatures and short exposure times may lead to incomplete synthesis [54,61]. The bioactive compounds from *Artemisia* extracts, especially proteins, flavonoids, and phenolic acids, affect NP size and stability by binding metal ions or reducing them to NPs [27,35,62]. Overall, nanoparticles synthesized from *Artemisia* extracts demonstrated a wide size variability depending on the metal type, plant species, and synthesis conditions.

### 3.2. Shape

Spherical morphology predominates among NPs synthesized using *Artemisia* extracts. However, cubic, hexagonal, triangular, polyhedral, and pentagonal shapes have also been reported. AgNPs exhibit morphological diversity, ranging from spherical [17,25] to cubic structures [57]. Basavegowda et al. reported spherical and triangular forms of AuNPs using *A. pallens* extract [53]. Metal oxide NPs, such as ZnONPs, MgONPs, and CuONPs, are typically spherical [46,49,63], whereas Fe_2_O_3_NPs have been observed in spherical and cubic morphologies [55,64]. As with NP size, morphology is influenced by several parameters, including pH, temperature, reactant concentration, and the phytochemical composition of the *Artemisia* extract.

### 3.3. Zeta Potential

Zeta potential (ζ) is a critical parameter in NP synthesis, influencing colloidal stability, surface interactions, and biological applications [65]. *Artemisia*-based NPs typically exhibit negative ζ values, indicating good colloidal stability. Reported ζ values range from −18.5 mV to −40.4 mV, with most clustering between −21 mV to −29 mV, corresponding to moderate stability [18,34,58,59,66,67,68,69,70]. Metal oxide NPs, such as ZnONPs (−38 mV) tend to display greater stability compared to metal NPs like AuNPs (−19.3 mV) [67,68]. Notably, AgNPs show a broader range of ζ values, from −18.5 mV to −40.4 mV, depending on synthesis conditions [34,59]. Key factors influencing ζ include pH, temperature, surface chemistry, and the composition of NPs. In alkaline conditions, ζ becomes more negative, whereas it becomes less negative in acidic environments [71]. Elevated temperature tends to decrease ζ values [72]. Additionally, Ardestani et al. [73] observed that NPs enriched in negatively charged functional groups exhibit more negative ζ values, thereby enhancing colloidal stability.

**Table 1:** Summary of synthesis, characterization, and potential applications of nanoparticles derived from extracts of *Artemisia* species.

Plant	Part Used	Solvent	Precursor	NPs	Characterization Techniques	Shape, Size and ζ	Phytoconstituents	Applications	Ref.
*Artemisia* *aucheri*	Aerial parts	Distilled water	Zn(NO_3_)_2_	ZnONPs	XDR, FTIR, FESEM, EDS	Spherical and granular particles; 15–40 nm (FESEM); ζ = −38 mV	C-N stretch, N-O stretch, N-O bond, N-H bond, alkynyl C≡C stretch, C-H stretch, O-H groups	Antibacterial activity	[67]
*Artemisia* (the species is not mentioned)	Leaves and stems	Distilled water	AgNO_3_ (1 mM)	AgNPs	FTIR, TEM	Spherical particles; 0.12–13 nm (AgNPs from leaves extract), 0.54–24 nm (AgNPs from stem extract) (TEM)	C-H stretching vibrations, C≡C bonds, C=O stretching vibrations, -C=O bonds, ketones, aldehydes, carboxylic acids, hydroxyl group (O-H), C–O–C group, C–O stretching vibrations	Antibacterial activity	[25]
	Leaves	Distilled water	FeCl_3_ (0.01, 0.04, 0.07, 0.1 M)	Fe_2_O_3_NPs	UV–Vis, FTIR, XRD, SEM, EDAX	Cubic particles; 19.72 ± 3.62–24.66 ± 3.62 nm (SEM)	O–H group stretching vibration, methyl group, stretching vibrations of C=C, C–C, and C–O of the aromatic cycles, C–H and C–O stretching of alcohols, carboxylic acids, ester and ether groups, Fe–O stretching band, phenols	Antioxidant activity	[64]
	Leaves	Methanol	Zinc salt solution	ZnONPs	TEM, UV-Vis, SEM	Spherical particles; 17 nm (TEM)	-	Antimicrobial activity	[39]
	Leaves	Distilled water	CuSO_4_ (0.005 mM)	CuNPs	UV-Vis, FTIR, SEM, XRD, EDX	Spherical and irregular particles; 38.5–48.5 nm (SEM)	O–H stretches of phenols, N–H stretches of amines, C=O and C=C vibrations, C-O stretching of phenolic compounds	Antimicrobial activity	[74]
*Artemisia* *abrotanum*	Leaves	Hydroalcoholic solution	AgNO_3_ (2 mM)	AgNPs	UV-Vis, TEM, XRD, EDX	Spherical particles; 20–30 nm (TEM), ζ = −28.5 ± 0.5 mV	OH, and NH stretching frequencies, CH stretching mode of hydrocarbon moieties, carbonyl group of amidic compounds, stretching of the C-H bond adjacent to a quinone moiety, stretching of the C=C bond adjacent to the quinone system, stretching of the C=C bonds of the various coupled aromatic systems, COO- asymmetrical and symmetrical stretching vibrations	Antimalarial activity	[58]
	Herb	Distilled water	Mg(NO_3_)_2_	MgONPs	UV-Vis, FTIR, XRD, SEM, TEM	Spherical particles; ~10 nm (XRD and SEM); <10 nm (TEM)	O–H bond vibrations of hydroxy group, C=O bond vibrations (flavonoids), alkynes group, C–H bending vibrations of aromatic tertiary amine group, piperitone, davanone, linanool, 1,8-cineole, silphiperfol-5-en-3-ol A, germacrene D, phenolic acids, flavonoid aglycones	Antioxidant activity	[46]
*Artemisia* *absinthium*	Whole plant	Distilled water	AgNO_3_	AgNPs	SEM, TEM, EDS	Spherical particles; 46 nm (TEM)	Terpenoids, phenolics, tannins, flavonoids, alkaloids, polysaccharides, hydroxycinnamic acid derivatives, proanthocyanidins; OH, C=O, stretching vibrations, C=C stretching in aromatic rings	Antimicrobial and antioxidant activity	[32]
	Leaves	Deionized water	AgNO_3_ (2 mM)	AgNPs	TEM, UV-Vis,	5–80 nm (TEM)	Artemisinin, *α*-bisabolol, thymol, *β*-pinene, limonene, stretch C=O, C-O, C-C	Antifungal activity	[75]
	Whole plant	Deionized water	AgNO_3_ (20 to 0.62 mM)	AgNPs	UV-Vis, DLS, TEM, EDX	Spherical particles, 5–20 nm (TEM, EDX); ζ = −32.4–(−40.4) mV	Polyphenols	-	[59]
	Leaves	Distilled water	Zn(NO_3_)_2_·6H_2_O (10%)	ZnONPs	XRD, SEM, EDX, FTIR, UV-Vis	Spherical and elliptical particles; 18.77–24.39 nm (XRD)	Phenolics, alkaloids, saponins, flavonoids, stretching vibrations of OH, C=O and C-O, COO- group	-	[36]
	Leaves	Pure water	Na_2_PdCl_4_	Pd/Fe_3_O_4_ NPs	FE-SEM, TEM, elemental mapping, EDX, ICP-OES, VSM, FTIR	Spherical particles; 15–30 nm (FE-SEM); 20–40 nm (TEM)	C–C expanding, C–O expanding, N–H bending, C=O stretching, and O–H stretching	Catalytic activity	[76]
	Flowers	Deionized water	AgNO_3_ (1 mM)	AgNPs	UV-Vis, TEM, FTIR, SEM, XRD	Spherical particles; 10.17–37.9 nm (TEM)	-	Antioxidant activity	[30]
	Leaves	Deionized water	AgNO_3_ 2mM	AgNPs	UV-Vis, TEM, EDX, DLS	-	Flavonoids, terpenoids, alkaloids, phenolic acids	Antimicrobial activity, disruption of zoospore function, reduction of sporoangial and zoospore release	[21]
	Leaves and stems	Distilled water	FeCl_3_ (0.1M)	IONPs	XRD, SEM, TEM, FTIR	Quasi-spherical particles; 4.7 ± 0.8 nm (SEM); 2.9–3.1 nm (TEM)	O-H stretching vibration of hydroxyl group, C=O bonds of amino acid and esters	Cytotoxic activity	[52]
*Artemisia* *abyssinica*	Leaves	Ethanol	Cu(NO_3_)_2_.3H_2_O (0.01, 0.1, 0.3, 0.5, 1 M)	CuONPs	SEM-EDX, UV-Vis, FTIR, TGA/DTA, TEM, XRD	Spherical particles; 18.4–24.6 nm (TEM)	Alkaloids, flavonoids, saponins, terpenoids, phenols, carbohydrates, proteins, carboxylic acids, stretching and bending vibrational frequencies of phenolic –OH, bending C–H of methylene groups, stretching vibration of C=O of phenolic compounds, stretching vibrations of the Cu–O bond	Antimicrobial and antioxidant activity	[14]
	Leaves	Ethanol (50%)	Cu(NO_3_)_2_.3H_2_O Zn(NO_3_)_2_.4H_2_O (0.1 M)	ZCNPs	UV-Vis, SEM, EDX, XRD, FTIR, TGA	Spherical particles; 13.09 nm (SEM)	O–H stretching, C=O and C–H stretching, N–H and –O–C stretching vibrations of aromatic cycle, C≡C from terminal alkyl groups, Cu–O and Zn–O bonds of metal biomolecules	Anticancer and antioxidant activity	[23]
*Artemisia afra*	Leaves	Distilled water	AgNO_3_ (1 mM)	AgNPs	TEM, XRD, UV-Vis, FTIR, SEM	Spherical and cubic particles; 30.74 nm (TEM)	Flavonoids, terpenoids, phenolics, O-H, C-H, C=O, C-O stretching	Antimicrobial and antioxidant activity	[54]
	Leaves	Distilled water	AgNO_3_ (1 mM) ZnCl_2_ (0.4 M) FeCl_3_	AgNPs ZnONPs Fe_2_O_3_NPs	UV-Vis, TEM, SEM, EDX, SAED	Spherical AgNPs 12 nm; rod, plate and spherical shape of ZnONPs 31 nm; hexagonal and spherical Fe_2_O_3_NPs 31 nm	heptadecyl-trans-*p*-coumarate	Antimicrobial and anticancer activity	[55]
*Artemisia* *annua*	Leaves	Distilled/deionized water	AgNO_3_ (1 mM)	AgNPs	HR-TEM, SEM, EDX, UV-Vis, XRD	Spherical particles; 9–50 nm; (HR-TEM)	Flavonoids, alkaloids, terpenoids, C-H stretching vibrations of aromatic compounds, amide C=O carbonyl stretch group	Antibacterial activity	[17]
	Leaves	Distilled water	AgNO_3_ (1 mM)	AgNPs	UV-Vis, TEM, DLS	Spherical particles; 20–90 nm (TEM); 49.4 ± 3.9–46.16 ± 8.56 nm (DLS)	Sesquiterpene lactones (artemisinin)	Antibacterial activity	[50]
	Hairy roots	Water/ ethanol	AgNO_3_ (1 mM)	AgNPs	TEM, FTIR, XRD, SEM, EDX, UV-Vis	Spherical, oval, triangular particles; 5–100 nm (TEM)	Flavonoids, phenolic acids, terpenoids, hydroxyl (–OH), C–H, C=C, and C–O, stretching vibration	Antimicrobial activity	[26]
	Leaves	Deionized water	HAuCl_4_·3H_2_O AgNO_3_ (5 mM)	AuNPs AgNPs	UV-Vis, TEM, XRD, EDX, TGA, FTIR	AuNPs spherical and triangular shape 14–40 nm (TEM); AgNPs spherical shape 30–40 nm (TEM)	O-H stretching, protein binding, C-H stretching, carbonyl vibrations of protein amides, C-N stretching vibrations of aromatic amines, C-O-C stretching	Antibacterial activity	[53]
	Leaves	Ethanol	AgNO_3_ (2 mM)	AgNPs	TEM, FTIR	Spherical particles; 7–27 nm (TEM)	Phenols, proteins	Antifungal activity	[37]
	Hairy roots	Ethanol (70%)	HAuCl_4_ (2 mM) AgNO_3_ (1 mM)	AuNPs, AgNPs	UV-Vis, FTIR, TEM	Spherical particles; 20 nm (TEM)	-OH group, N-H stretch, stretching vibration of -C=O, stretching vibration of carboxylate -COO- groups, -CH_3_ groups	Photocatalytic activity	[27]
	Hairy roots	Ethanol (70%)	HAuCl_4_	AuNPs	TEM	1–100 nm (TEM)	-	-	[38]
	Callus	Distilled water	AgNO_3_ (1 mM)	AgNPs	TEM, AFM, XRD, FTIR	Spherical particles; 2.1–45.2 nm (TEM)	NH stretching, C-H bonds, C=O stretching bands, C=C stretching, C-C stretching, C-O stretching	Antibacterial activity	[31]
	Leaves	Distilled water	AgNO_3_ (0.1–0.2 M)	AgNPs	UV-Vis, FTIR	1–5 nm (DLS); ζ = −26.1 mV	O-H phenolic group, C=C aromatic stretching, N-H secondary amide stretching, C-H methylene group stretching, C-N stretching of aromatic amines	Antimicrobial, antioxidant, dye degradation activity	[18]
	Stems and leaves	Ethanol and hydroethanolic solution	Zn(CH_3_COO)_2_·2H_2_O	ZnONPs	UV-Vis, XRD, fluorescence spectroscopy, SEM-EDS	Spherical particles; 21.34–24.71 nm (XRD)	-	-	[63]
	Leaves	Triple distilled water	AgNO_3_ (1 mM)	AgNPs	UV-Vis, TEM, SAED, DLS	Spherical particles, 20–90 nm	-	Antimicrobial activity	[50]
*Artemisia* *arborescens*	Leaves	Hydroalcoholic solution	AgNO_3_	AgNPs	UV-Vis, FTIR, TEM	Spherical particles; <50 nm (TEM)	Amines, proteins, phenolic compounds, OH, and NH stretching frequencies, sp3 CH stretching mode of hydrocarbon moieties	Anticancer and apoptosis- inducing activity	[19]
	Leaves	Hydroalcoholic solution	AgNO_3_ (2 mM)	AgNPs	UV-Vis, TEM, XRD, EDX	Spherical particles; 20–30 nm (TEM); ζ = −29 ± 1 mV	OH and NH stretching frequencies, CH stretching mode of hydrocarbon moieties, carbonyl group of amidic compounds, stretching of the C-H bond adjacent to a quinone moiety, stretching of the C=C bond adjacent to the quinone system, stretching of the C=C bonds of the various coupled aromatic systems, COO- asymmetrical and symmetrical stretching vibrations	Antimalarial activity	[58]
*Artemisia* *campestris*	Leaves and stems	Distilled water	AgNO_3_ (2.5 mM)	AgNPs	FESEM, EDS, FTIR, SEM, spectrophotometry	Spherical particles; 63–68 nm (SEM)	Alkenes functional groups, -OH group	Antimicrobial activity	[77]
*Artemisia* *capillaris* Thunberg	Aerial parts	Water/ethanol (70%)	AgNO_3_ (1 mM)	AgNPs	UV-Vis, HR-TEM, AFM	29.71 nm for water extract; 29.62 nm for 70% ethanol extract (AFM)	Flavonoids aliphatic carbonyl (-C=O) stretching, aromatic C=C stretching vibration, alkene C=C stretching	Antibacterial activity	[78]
*Artemisia* *carvifolia* Buch	Shoots	Distilled water/ methanol	AgNO_3_ (5 mM)	AgNPs	FTIR, UV-Vis, SEM, XRD, EDX	Polyhedral particles; 80 ± 6 nm (SEM)	Flavonoids, phenolics	Anticancer activity	[35]
*Artemisia chamaemelifolia*	Aerial parts	Ethanol (70%)	Na_2_SeO_3_ (10 mM)	SeNPs	SEM, TEM, DLS	Spherical particles; 110 nm (TEM)	Carbonyl group (C=O), hydroxyl group (-OH), Se-O bond	Anticancer, antibacterial activity	[42]
*Artemisia* *ciniformis*	Leaves	Distilled water	AgNO_3_ (0.01 mM)	AgNPs	TEM, SEM, UV-Vis, FTIR, EDX, DLS	Spherical particles; 4–14 nm (TEM); ζ = −24.33 ± 3.05 mV	Alcohols, phenols, alkaloids, tannins, terpenes and terpenoids, stretching vibrations of the O-H and N-H groups in the extracted alcoholic, phenolic and aminic compounds, C=C bond of alkenes and aromatic rings, stretching vibration of alcoholic C-O bonds	Anticancer activity	[41]
*Artemisia deserti*	Leaves	Deionized water	CuSO_4_ (0.01 M)	CuONPs	UV-Vis, FTIR, TEM, FESEM, EDX, XRD, DLS, PDI	Spherical particles; 9.72 ± 7.80 nm (TEM); ζ = −17.1 ± 2.8 mV	Stretching vibration frequencies of alcohol and polysaccharide O–H group, –H stretching vibration of amide group of proteins, stretching vibration of C-H aliphatic compounds, C=O carbonyl group in amides, C=C bonds in terpenes, C-O bonds of alcohol and ether compounds, C-C-H bonds of terpenoids	Anticancer activity	[51]
*Artemisia haussknechtii*	Leaves	Distilled water	AgNO_3_, CuSO_4_, TiO (OH)_2_ (0.001, 0.01, 0.1 M)	AgNPs, CuNPs, TiO_2_NPs	UV-Vis, XRD, FTIR, FE-SEM, AFM	Triangle AgNPs 10.69 ± 5.55 nm; Spherical CuNPs 35.36 ± 44.4 nm; Spherical TiO_2_NPs 92.58 ± 56.98 nm (SEM)	N–H stretch, aromatic C=C bending, –C–H bending, C-O stretch (alcohol), =C-H bending, C-Cl stretch (alkyl-halide), C-Br stretch, N-H stretch (amide), C=C stretch (aromatic), C-F stretch group	Antioxidant and antibacterial activity	[49]
*Artemisia herba-alba*	Leaves	Deionized water	AgNO_3_ (0.1 M)	AgNPs	UV-Vis, TEM, XRD, FTIR	Spherical particles; 9.68–36.7 nm (TEM)	Stretching vibrations of N–H, C–H of benzene rings and carboxylic acids, C-C stretching vibrations of aromatic amines, C≡C bond, C–O stretching vibrations of alcohol/ether, C=C amide I group bonds	Insecticide activity	[79]
	Leaves	Distilled water	AgNO_3_ (2–5 mM)	AgNPs Ag_2_ONPs	UV-Vis, ATR-FTIR, XRD, SEM, TG-DTA	Spherical particles; 150–250 nm (SEM)	-O-H and -C=O groups, N-H bending vibrations of the primary amides group	-	[15]
	Leaves	Ethyl alcohol (70%)	-	ZnONPs	TEM, XRD	Quasi-spherical particles; 25 ± 5 nm (TEM)	-	Cardioprotective properties	[80]
	Leaves	Distilled water	Ni(NO_3_)_2_·6H_2_O (98%)	NiONPs	UV-Vis, FTIR, XRD	Spherical particles; 7.49 ± 0.0005 nm (0.01 M precursor); 8.58 ± 0.0001 nm (0.025 M precursor); 8.13 ± 0.0001 nm (0.05 M precursor); 10.7 ± 0.0002 nm (0.075 M precursor)	O–H stretching vibration mode, bond of C-C or C–N stretching vibration mode, stretching vibrations of C=C of aromatic compounds, C=O stretching of carboxylic acids, C–O stretching vibration mode	Antioxidant activity	[81]
*Artemisia indica*	Leaves	Methanol	AgNO_3_ (0.1 M)	AgNPs	UV-Vis, TEM, Nano Zeta sizer, PXRD, TGA, FTIR	Spherical and oval particles; ~20 nm (TEM); ζ = −23.4 mV	O–H stretching indicating phenolic type of compounds	Antibacterial and cytotoxic activity	[47]
*Artemisia* *judaica*	Aerial parts	Distilled water	AgNO_3_ (1 mM)	AgNPs	UV-Vis, TEM	-	Phenols, flavonoids	Antitrichinellosis activity	[28]
*Artemisia* *kopetdaghensis*	Shoots	Distilled water	AgNO_3_ (1 mM)	AgNPs	UV-Vis, FTIR, XRD, TEM	Spherical particles; 3–35 nm (TEM)	O–H stretching vibration, C–H vibration (stretching) of aromatic compounds, C–N and C–C stretching of proteins, nitro N–O bending, C–O stretching of phenols and alcohols	Anticancer, antimicrobial, and dye degradation activity	[82]
*Artemisia* *lerchiana*	Leaves	Distilled water	AgNO_3_	AgNPs	UV-Vis, XRD	24.83 nm (XRD)	-	-	[83]
	Leaves	Distilled water	AgNO_3_	AgNPs	TEM, FTIR, UV-Vis, AFM	Spherical particles; 4–19 nm (TEM)	C=O stretching, C=C stretching of alkynes, O–H stretching, N–H stretching vibrations	-	[3]
*Artemisia* *marschalliana* Sprengel	Aerial parts	Distilled/deionized water	AgNO_3_ (0.01 mM)	AgNPs	UV-Vis, XRD, FT-IR, TEM, FE-SEM, EDS	Spherical particles; 5–20 nm (TEM); ζ = −31 mV	Essential oils, flavonoids, phenolic acids; O-H, C-H, C=O, C-O, C-N, C-O-C stretching vibrations	Anticancer and antibacterial activity	[73]
*Artemisia* *monosperma*	Aerial parts	Deionized water	AgNO_3_ (10^−3^ M)	AgNPs	UV-Vis, TEM, infrared spectra, FTIR	Spherical and irregular particles; 13–18 nm (TEM)	OH groups, C-H stretching vibration, C-O and C=O stretching of the carbonyl functional group, C=O of the amide groups of protein, -C-C- stretching vibrations of aromatic rings	Antimicrobial activity	[84]
*Artemisia* *nilagirica*	Leaves	Distilled water	AgNO_3_ (1–6 mM)	AgNPs	SEM, EDX	Square particles; 70–90 nm (SEM)	-	Antimicrobial activity	[85]
*Artemisia* *oliveriana*	Aerial parts	Distilled water	AgNO_3_	AgNPs	UV-Vis, FTIR, TEM, SEM	Spherical particles; 10.63 nm (SEM, TEM)	Ether compounds (C–O–C), alkane C–H stretching vibrational, N–H stretching vibration of the amine, protein binding	Antioxidant, antimicrobial and anticancer activity	[86]
*Artemisia* *pallens*	Leaves	Distilled water	MgCl (0.15 M)	MnNPs	UV-Vis, SEM, EDX, XRD, FTIR	Octahedral particles; 275 nm (SEM)	O-H stretching of phenols and alcohols, C=O stretching	Antibacterial and anticancer activity	[87]
	Leaves	Distilled water	Zn_2_·6H_2_O	ZnONPs	XRD, SEM, EDX, TEM UV-Vis	Hexagonal particles; 50–100 nm (TEM)	asymmetric C-H stretching, symmetrical stretching of methylene (CH2-S), C-O-H bending band, CH_2_ group, -CO-O-CO-C stretching	Antimicrobial activity	[22]
	Leaves	Distilled water	AgNO_3_ (1 mM)	AgNPs	UV-Vis, FTIR, XRD, SEM, EDS	Spherical particles; 25–35 nm (SEM)	Terpenoids, flavonoids, C=C stretching vibration in aliphatic compounds, CH_2_ and CH_3_ groups, -O groups of polyols	Antimicrobial activity	[88]
*Artemisia* *quttensis* Podlech	Aerial parts	Ethanol (50%)	AgNO_3_ (0.01 mM)	AgNPs	UV-Vis, XRD, FE-SEM, TEM, EDS, FTIR	Spherical particles; 5–25 nm (TEM)	Phenolic, alkaloids compounds, carbohydrates, amino acids, O–H stretching vibration of phenols, alkane C–H stretching vibrations, amide I vibrations, amine groups, C–H stretching of Aldehydic, C–O stretching vibration	Antioxidant, antibacterial and anticancer activity	[29]
*Artemisia schrenkiana*	Leaves	Distilled water	AgNO_3_ (1 mM)	AgNPs	UV-Vis, TEM, SEM, XRD, EDX	Spherical particles, 0.60–1.65 nm	Flavonoids, polyphenolic compounds, organic acids, coumarins	Anticancer, antioxidant activity	[24]
*Artemisia* *scoparia*	Stems and Leaves	Deionized water	AgNO_3_ (3 mM)	AgNPs	UV-Vis, FTIR, TEM, SEM, DLS	Spherical particles; ~43.30 nm (FESEM); ζ = −25.3 mV	Hydroxyl -OH group, nitrile C=N, C=O, amide carbonyl group	Antimicrobial and catalytic activity	[70]
*Artemisia sieberi*	Leaves	Distilled water	AgNO_3_ (1 mM)	AgNPs	TEM, UV-Vis, XRD, FTIR	Spherical particles; 8–22 nm (TEM)	O–H stretches of carboxylic bands, carbonyl stretching bands, alkene groups	Antifungal activity	[16]
	Shoots	Distilled water	AgNO_3_ (2 µM)	AgNPs	UV-Vis, TEM, FTIR, DLS	Spherical particles; 27.5 ± 3.5 nm (TEM)	O-H, C≡C, C-H, N-O, C-O, C=C, C-X stretching vibrations	Antimicrobial activity	[33]
	Leaves	Distilled water	AgNO_3_ (1 mM)	AgNPs	UV-Vis, SEM, XRD, FTIR, TEM	Cubic particles, 20 nm (XRD); Spherical and cubic particles 20 nm (TEM); Spherical particles, 41–88 nm (SEM)	O-H stretching vibrations, -C-H- stretching of alkenes, C=O stretching vibrations or stretching of alkenes; -C-N- stretching vibrations of amines, -C-O stretching of alcohols, ethers, carboxylic acids and anhydrides	-	[57]
*Artemisia* *stelleriana*	Leaves	Distilled water	AgNO_3_ (0.01 M)	AgNPs, AgONPs	UV-Vis, XRD, FTIR, EDX, FESEM	Spherical particles; 22.7 nm (FESEM)	Flavonoids, tannins, alkaloids, monoterpenes and sesquiterpenes; O-H, N-H, C=C, CO-O-CO, C-Br, C≡C, N-O, C=C=C, C-F stretching vibrations	Antioxidant and photocatalytic activities	[20]
	Leaves	Distilled water	Zn(CH_3_COO)_2_·2H_2_O (0.1 M)	ZnONPs	UV-Vis, FTIR, FESEM, EDX, XRD	Near-spheroid, thin rod and irregular; 10–100 nm (SEM); 22.54 nm (XRD)	C=C bending, O-H bending	Photocatalytic activity	[45]
*Artemisia tilesii*	Hairy roots	Water/ethanol	AgNO_3_ (1 mM)	AgNPs	TEM, FTIR, XRD, SEM, EDX, UV-Vis	Spherical, oval, triangular particles; 5–100 nm (TEM)	Flavonoids, phenolic acids, terpenoids, hydroxyl (–OH), C–H, C=C, and C–O, stretching vibration	Antimicrobial activity	[26]
*Artemisia* *turcomanica*	Leaves	Ethanol	AgNO_3_ (0.01 mM)	AgNPs	TEM, SEM, UV-Vis, FTIR, XRD	Spherical particles; 22 nm (SEM, TEM)	Phenolic and alcoholic O–H groups, aromatic group C–H of benzene rings and aliphatic groups, C=O bond of the carbonyl amide protein group, C–O–C phenolic stretching vibration, C–O–H stretching vibrations	Anticancer activity	[89]
*Artemisia* *vulgaris*	Leaves	Deionized water	AgNO_3_	AgNPs	UV-Vis, SEM, EDX, TEM, AFM, FTIR	Spherical particles; 27–53 nm (SEM); 25 nm (TEM)	terpenes, essential oils, coumarins, sterols, flavones, polyphenols, *α*-thujone, *α*-terpineol, geraniol, caryophyllene, O–H stretching of the phenolic group, the carbonyl stretching of –C=O, aromatic stretching of –C–N and –C–O or –C–O–C–	Antimicrobial activity	[90]
	Aerial parts	Distilled water	AgNO_3_ (1 mM)	AgNPs	UV-Vis, SEM, FTIR	Spherical particles; 30 nm (SEM)	Amino acids, proteins, carbohydrates, glycosides, phenolics, steroids, terpenoids, O-H stretching of alcohols, N-H stretching of amines, C–H of alkanes, C=O of carboxylic acid/aldehydes or ester, N–C=O amide I bond of proteins, H–C=O: C–H stretch of aldehydes, C≡N stretch of nitriles, N–O of nitro compounds, C–N of aliphatic amines or alcohol/phenol, N–H deformation of amines, and C–C bending	Antimicrobial activity	[91]
	Leaves	Distilled water	SnCl_2_. 2H_2_O (0.05 M)	TONPs	UV-Vis, FTIR, XRD, SEM, EDX	Spherical particles; 48.76 nm (XRD)	–OH groups, stretching vibrations of aliphatic C–H groups, Sn–O, O–Sn–O bond	Antifungal activity	[92]
	Leaves	Distilled water	AgNO_3_ (1 mM)	AgNPs	SEM, EDX	Spherical particles; 7 nm (XRD)	O-H stretching vibration of alcohol and phenol, C-H stretching vibration of aromatic compounds, C-C and C-N stretching vibrations of amines, N-H vibration, C-OH stretching vibrations of phenols	Catalytic and metal-sensing activity	[48]
	Leaves	Distilled water	AgNO_3_ (1 mM)	AgNPs	UV-Vis, SEM, AFM, FTIR	Spherical particles	O-H stretching vibration of alcohol and phenol, C-H stretching vibration of aromatic compounds, C-C and C-N stretching vibrations indicates the presence of proteins, C-OH stretching vibrations of the phenols, amine C-N stretching	Catalytic activity	[43]
	Leaves	Methanol (99.8%)	AgNO_3_ (0.01 M)	AgNPs	UV-Vis, FESEM, FTIR, XRD	∼28 nm (XRD)	O-H bond stretching, C–H stretching of alkane, C=O or C=C stretching of carbonyl compounds, C-N stretching of amine	Antimicrobial activity	[44]
	Leaves	Methanol (99.8%)	Cu(NO_2_)_2_·3H_2_O Zn(NO_3_)_2_·6H_2_O	CuONPs; ZnONPs; CuO-ZnO NPs	UV-Vis, FTIR, XRD, FESEM, EDS, elemental mapping	CuONPs: 17.24 nm (XRD) ZnONPs: 20.74 nm (XRD) CuO@ZnO NPs: 22.5 nm (XRD)	O-H bond stretching, C–H stretching of alkane, C-N stretching, –C-O and –C-O-C- stretching of ester and tertiary alcohol, C=C stretching of alkenes and stretching modes of nitrate anions, C=O stretching	Photocatalytic activity	[40]
	Leaves	Distilled water	AgNO_3_ (0.01 M)	AgNPs	UV-Vis, TEM, FTIR	2.90–200 nm (TEM)	O-H stretching of alcohol, N-H stretching vibration, N-H stretching of the secondary amide of the protein, O-H phenolic group, stretch vibration of -C=C-, C-N and C-C stretching (aromatic), N-H and C-N (amines) stretch vibration of the proteins, C-OH of the phenols, flavonoids, triterpenoids	Antimicrobial activity	[60]
	Leaves	Distilled water	HAuCl_4_ (1 mM)	AuNPs	UV-Vis, TEM, XRD, DLS, FTIR, EDX, ZP	Spherical, triangular and hexagonal particles; 50–100 nm (TEM); 89.76 nm (DLS) ζ = −19.3 mV	O-H stretching Alcohol, C-H stretching alkene, C-H stretching aldehyde, N-h bend amine, O-H bend phenol, C-N stretching aromatic amine, C-O stretching alkyl-ether	Larvicidal activity	[68]
	Leaves	Deionized water	AgNO_3_ (1 mM)	AgNPs	UV-Vis, SEM, Zeta sizer	Spherical particles; 190.8 nm (Zeta sizer); ζ = −18.5 mV	-	Antifungal activity	[34]
Russian *Artemisia*	Leaves	Distilled water	Zn(NO_3_)_2_ (0.03 mM), gold solution (0.06 mM)	Au–ZnO NCs	UV-Vis, EDS, XRD, TEM, FTIR, FESEM	Spherical particles; 40 ± 5 nm (FESEM, TEM)	O-H of alcohol and phenol, C=O stretch, C-H bending vibrations	Cytotoxic activity	[61]

## 4. Artemisia-Based Nanoparticle Applications

### 4.1. Antimicrobial Activity

The findings indicate that nanoparticles synthesized from extracts of various *Artemisia* species exhibit significant antimicrobial properties against a wide array of microorganisms. Specifically, AgNPs [17,32,44,54,88], AuNPs [53], ZnONPs [55], SeNPs [42] and CuNPs, TiO_2_NPs [49], CuONPs, manganese nanoparticles (MnNPs) [87], and iron oxide nanoparticles (Fe_2_O_3_NPs) [55] demonstrated antibacterial activity against both Gram-positive bacteria (e.g., *Staphylococcus aureus*, *Bacillus subtilis)* and Gram-negative bacteria (e.g., *Escherichia coli*, *Pseudomonas aeruginosa*). Furthermore, antifungal effects against species such as *Candida albicans* and *Aspergillus flavus* were observed [39,60,85].

Analyses have revealed that AgNPs synthesized from *A. aucheri* [67], *A. annua* [50], *A. tilesii* [26], *A. vulgaris* [60], *A. nilargirica* [85], *A. absinthium* [32], and *A. marschalliana* [73] extracts demonstrated a higher inhibitory effect against Gram-positive bacteria than Gram-negative strains. The investigation conducted by Kobylinska et al. clarified that *A. annua*- and *A. tilesii*-based AgNPs exhibited a significantly enhanced inhibitory outcome against *S. aureus* compared to *E. coli*. This difference may be attributed to the small size of AgNPs (1–20 nm), which increased their capacity to disrupt the cell membrane by increasing the likelihood of interaction with a larger surface area of the plasmalemma [26]. Similarly, Alomari and Rasheed noted that AgNPs synthesized using *A. vulgaris* extract yielded the highest antibacterial activity against *S. aureus*, with an inhibition zone (IZ) of 15 mm and 18 mm, respectively. In contrast, the IZ against *E. coli* measured approximately 13 mm in both evaluations [60,90].

In contrast, Aghajanyan et al. [50] revealed that *A. annua*-based AgNPs exhibited a greater antibacterial performance against Gram-negative strains at a concentration of 100 μg/mL. This effect could be attributed to the presence of sesquiterpene lactones in *A. annua* or the small size of the nanoparticles [50]. Similarly, Vijayakumar et al. [85] reported that *A. nilagirica*-mediated AgNPs displayed the highest effectiveness against Gram-positive bacteria, with an IZ of nearly 3.0 mm, compared to approximately 2.0 mm against Gram-negative bacteria.

A study comparing the antimicrobial activity of pure *A. absinthium* extract and its AgNPs using the disc diffusion method revealed a considerable difference in efficacy. While the pure extract inhibited six out of 15 bacterial strains (IZ ranged from 3.0 mm for *E. coli* to 8.4 mm for *A. johnsonii*), the AgNPs inhibited growth in all the tested strains, with IZ values ranging from 9.0 mm for *E. cloacae* to 19.3 mm for *C. sakazakii* [32]. The *A. kopetdaghensis* AgNPs demonstrated potent antibacterial activity, significantly outperforming the *A. kopetdaghensis* aqueous shoot extract. The largest inhibition zones were observed against *K. pneumoniae* (22.3 mm) and *S. aureus* (17.8 mm) [82]. Likewise, Achamo et al. [14] tested the pure extract of *A. abyssinica* and its CuONPs using diffusion and dilution (MIC) methods. The results showed that the *A. abyssinica* extract had higher antibacterial ability than *A. absinthium* extract in inhibiting the *S. aureus*, *P. aeruginosa,* and *E. coli*. Additionally, CuONPs inhibited bacterial growth at all tested concentrations and demonstrated better efficacy than AgNPs [14]. The smaller size of the CuONPs (24.6 nm) may provide an advantage, allowing them to penetrate the cellular membrane more efficiently than the AgNPs [93]. The antimicrobial effectiveness of biosynthesized ZnONPs was tested on clinical strains of *P. aeruginosa*, revealing significant activity. The ZnONPs exhibited bactericidal effects on the isolates at concentrations ranging from 3.125 to 100 μg/mL [39]. Additionally, ZnONPs obtained using *A. absinthium* extract exhibited higher antibacterial activity against *S. aureus*, *B. subtilis*, and *P. aeruginosa* than pure ZnONPs.

The synthesized AuNPs with *A. annua* extract displayed superior activity against *E. coli* and *B. cereus* compared to AgNPs [53]. Park et al. [78] examined the antibacterial properties of AgNPs obtained from both aqueous and 70% ethanol extracts of *A. capillaris,* revealing that the MIC values ranged from 8.35 to 16.7 μg/mL. Notably, the AgNPs reduced by 70% ethanol extract registered lower MIC values against Gram-negative strains [78]. Moreover, the volume ratio of plant extract to metal salt solution (*v*/*v*) affects the antimicrobial efficacy of the synthesized NPs. *A. vulgaris*-based AgNPs prepared at a 1:5 ratio exhibited higher antimicrobial activity than those synthesized at a 1:3 ratio [44].

Among the biosynthesized NPs, CuONPs obtained using *A. abyssinica* extract exhibited significant antifungal activity, particularly against *A. flavus*, with the highest IZ recorded at 25 mm and an MIC of 15 μg/mL [14]. The *A. vulgaris*-based TONPs were effective in inhibiting biofilm formation and disrupting mature biofilms of *C. albicans* [92]. Additionally, *A. pallens*-mediated AgNPs demonstrated significant antibiofilm activity against *P. aeruginosa*, *S. mutans*, and *S. aureus* [87].

AgNPs synthesized using extracts of *A. annua*, *A. tilesii*, *A. afra*, *A. Absinthium*, and *A. Vulgaris* displayed remarkable restraining effects against *Candida albicans*, *Phytophthora* species, and *Aspergillus fumigatus* [16,21,26,34,54]. Contrarily, one study mentioned that *A. vulgaris*-synthesized AgNPs showed no antifungal activity against *C. albicans* and *A. flavus* [60]. The antifungal potential of *A. absinthium*-based AgNPs was tested using a 96-well microtiter plate format against various *Phytophthora* species. The results showed that AgNPs restrained mycelial growth, zoospore germination and production, and germ tube elongation [21].

### 4.2. Antioxidant Activity

Numerous studies have evaluated the antioxidant activity of nanoparticles, which can enhance their potential applications in various fields, including medicine and food preservation. Most research has focused on the antioxidant potential of AgNPs synthesized using *A. afra*, *A. annua* L., *A. oliveriana* and *A. absinthium* extracts [18,32,54,86], as well as CuNPs derived from *A. haussknechtii* and *A. abyssinica* [14,49]. In contrast, fewer articles have assessed the reducing power of biosynthesized Fe_2_O_3_NPs [64], MgONPs [46], TiO_2_NPs [49], and other types of nanoparticles.

MgONPs derived from *A. abrotanum* displayed higher antioxidant activity, with an IC50 value of 4.73 μg/mL, compared to the pure plant extract, which had an IC50 of 6.28 μg/mL [46]. Additionally, AgNPs from *A. vulgaris* extract reduced DPPH radicals by approximately 80%, while the pure leaf extract registered around 50% reducing capacity [90]. Achamo also reported that CuNPs produced from *A. abyssinica* effectively reduced DPPH radicals by about 89% with an IC50 value of 5.75 μg/mL [14]. Both *A. absinthium* extract and its AgNPs displayed similar results in FRAP, DPPH, and CUPRAC assay methods. However, AgNPs showed slightly higher antiradical activity in the ABTS method (0.55 ± 0.05 mmol/g DW) and TEPH method (0.057 ± 0.005 mmol/g DW) [32]. Furthermore, AgNPs synthesized from *A. marschalliana* Sprengel extract reduced DPPH free radicals by up to 61% [73], while AgNPs from *A. stelleriana* extract registered the highest antioxidant activity at 600 μg/mL, achieving a 76.3% reduction [20]. Also, AgNPs from *A. quttensis* diminished the DPPH free radicals by 65.04% at a concentration of 450 μg/mL [29]. Using the DPPH and FRAP method, Orshiso et al. demonstrated that bimetallic ZnO-CuO nanoparticles reduced DPPH radicals by 95.71% and reduced ferric ions with an absorbance of 1.826 at 200 μg/mL [23]. Another study comparing the antioxidant performance of green-synthesized AgNPs, CuNPs, TiO_2_NPs and *A. haussknechtii* leaf extract demonstrated that AgNPs exhibited the highest DPPH free radical scavenging activity, achieving 89.02%. In comparison, CuNPs, TiO_2_NPs, and the leaf extract showed scavenging efficiencies of 74.45%, 68.43%, and 67.54%, respectively [49]. Similarly, the antioxidant activity of Fe_3_O_4_NPs synthesized using *Artemisia* sp. was evaluated using multiple assays. Total antioxidant capacity (TAC) analysis yielded a value of 89.231 ± 20.456 mg ascorbic acid equivalents (AAEs)/g NPs at a precursor concentration of 0.1 M. The FRAP assay indicated that the 0.7 M sample exhibited the highest reducing capacity, corresponding to 438.330 ± 46.995 mg FeSO_4_ equivalents/g NPs. Furthermore, the lowest IC_50_ value, indicating the strongest antioxidant activity, was recorded for the 0.1 M sample (0.782 ± 0.025 mg/mL) [64].

The concentration of the metal precursor was found to significantly influence the antioxidant activity of the synthesized NPs. For instance, *A. herba-alba*-mediated NiONPs synthesized using Ni(NO_3_)_2_ at molar concentrations of 0.01, 0.025, 0.05, and 0.075 M exhibited peak antioxidant efficacy at different concentrations depending on the assay employed. The 0.01 M NiONPs showed the highest DPPH radical scavenging activity, achieving an inhibition rate of 78.26%, and the lowest IC_50_ value (5.989 mg/mL). In contrast, nanoparticles synthesized at 0.05 M and 0.025 M demonstrated a superior total antioxidant capacity and reducing power, respectively. These distinct performance maxima suggest that variations in precursor concentration directly modulate the nanoparticles’ ability to neutralize free radicals and reduce oxidative species [81].

### 4.3. Anticancer Activity

Various studies have explored the anticancer activities of different types of biosynthesized NPs. For instance, AgNPs demonstrated a pronounced antiproliferative effect against the tested cancer lines, HeLa and MCF-7, and significantly induced apoptosis in both cancer lines [19]. Furthermore, the safety profile of *A. turcomanica*-mediated and commercial AgNPs was evaluated using normal L-929 cells and AGS gastric cancer cells. In AGS cells, treatment resulted in dose-dependent growth inhibition, with biogenic AgNPs exhibiting superior potency compared to commercial AgNPs, with IC_50_ values of 4.88 µg/mL and 6.37 µg/mL, respectively, achieving 95% inhibition at 100 µg/mL relative to 84%. On normal L-929 cells, while 100 µg/mL significantly reduced cell proliferation, lower concentrations (3 and 5 µg/mL) maintained high cell viability (>92%) comparable to the control group. Notably, the IC_50_ values for L-929 cells were 14.56 µg/mL (biogenic) and 15.43 µg/mL (commercial). These values are markedly higher than those observed for the AGS cancer line, indicating a selective toxicity toward cancer cells [89]. In contrast, AgNPs synthesized from *A. quttensis* recorded similar IC_50_ values for HT29 colon cancer (58.18 μg/mL) and normal cell lines (59.97 μg/mL), demonstrating no preference between cell types [29]. In addition, AgNPs derived from *A. afra* extract displayed non-selective cytotoxicity against MCF7 (breast adenocarcinoma) and A549 (human lung adenocarcinoma) cancer cells and normal HEK293 cells, while ZnONPs synthesized using the same plant extract did not exhibit any antiproliferative activity [55].

AgNPs derived from *A. schrenkiana* extract exhibited excellent biocompatibility and selective toxicity, with significantly higher IC_50_ values for normal liver cells (10 µg/mL) compared to liver cancer cells (2.5 µg/mL) [24]. SeNPs from *A. chamaemelifolia* extract displayed dose- and time-dependent cytotoxicity against both HT-29 colon cancer cells and HEK293 normal kidney cells, with concentrations of 100 μg/mL significantly reducing viability after 24–48 h. Notably, biosynthesized SeNPs demonstrated superior cytotoxicity compared to chemically synthesized counterparts or *A. chamaemefolia* leaf extract alone, attributable to the synergistic interaction between selenium cores and plant-derived capping agents on the nanoparticle surface. Additionally, results showed that pro-apoptotic genes such as Bax, Caspase3, and Caspase9 were upregulated in HT-29 cancer cells [42]. Biogenic Fe_2_O_3_NPs exhibited cytotoxic activity against the A375 cell line when administered at a concentration of 500 µL/mL. Furthermore, this toxicity was found to be time-dependent, with cell viability decreasing as the duration of exposure increased. In contrast, biogenic Fe_2_O_3_NPs demonstrated low cytotoxicity toward normal HaCaT cells, maintaining cell viability above 80%, indicating relative selectivity against cancer A375 cells [52]. Phyto-mediated CuONPs synthesized using *A. deserti* extract decreased the cell viability of A2780 cells (IC_50_ = 13.17 μg/mL) more efficiently than cisplatin (IC50 = 172.18 μg/mL). However, on normal human foreskin fibroblast (HFF) cells, these NPs exhibited significantly lower cytotoxicity than cisplatin, being approximately 47 times less toxic. The green-synthesized CuONPs actively drive apoptosis in cisplatin-resistant A2780-CP ovarian cancer cells by significantly upregulating pro-apoptotic genes (Bax, Caspase3, Caspase9, and p21) while simultaneously downregulating the anti-apoptotic gene *Bcl2*. Furthermore, the nanoparticles suppress cell division by downregulating cyclin B and cyclin D [51].

The safety and biocompatibility of *A. indica*-mediated AgNPs were tested on normal fibroblast cells (L929). The results showed a cell viability of 83%, indicating that the AgNPs are compatible and non-toxic [47]. *A. pallens*-based MnNPs demonstrated cytotoxic activity against cancer cell lines A431, A549 and MCF7, with the highest inhibition observed in A431 cells (IC50 = 25 μg/mL). In these cells, AP-MnNPs induced ROS generation, mitochondrial disruption, DNA breakage, and G2/M arrest. Furthermore, they triggered apoptosis by upregulating Bax and caspase-3 while downregulating Bcl2, ultimately suppressing the PI3K/Akt signaling pathway [87]. Additionally, *A. kopetdaghensis*-based AgNPs demonstrated potent cytotoxicity against the HepG2 cell line, which increased with both exposure time and concentration [82].

### 4.4. Agriculture and Food Safety

The integration of NPs in agriculture and food safety holds promise for enhancing sustainability and efficiency. The AgNPs derived from *A. annua* extract demonstrated positive activity in seed germination, achieving a germination percentage of 43%. They also exhibited effective degradation activity against food dyes [18] and extended the vase life of *D. caryophyllus* flowers by 3.12 days [31]. Furthermore, Ali et al. [21] found that AgNPs protected tobacco plants against *Phytophthora* infection. After treatment, the survival percentage was 96.3% at 100 μg/mL concentration and 77.8% at 10 μg/mL concentration. Foliar application of *A. absinthium* AgNPs improved salt tolerance in basil, significantly enhancing biomass, phenol content, and antioxidant enzyme activity (CAT, APX). The treatment mitigated stress, whereas untreated plants exhibited elevated proline and malondialdehyde levels [30].

### 4.5. Environmental Remediation

The use of NPs in environmental remediation offers promising solutions to pollution and contamination challenges. For instance, AgNPs synthesized using *A. stelleriana* extract have demonstrated significant photocatalytic activity, degrading reactive blue 222A (RB-222A) and RB-220 dyes with efficiencies of 90.8% and 94.6%, respectively, within 80 min [20]. Additionally, *A. annua*-mediated AuNPs and *A. vulgaris*-mediated AgNPs efficiently degraded methylene blue (MB) after increasing time exposure [27,43]. Furthermore, Dobrucka demonstrated the catalytic properties of MgONPs obtained from *A. abrotanum* against methyl orange (MO), and Adhikari et al. found that AgNPs by *A. vulgaris* could transform 4-nitrophenol to 4-aminophenol within 8 min and could be used as a detector for Hg^2+^ ions [46,48]. Moreover, an *A. vulgaris*-based AgNP solution visibly changed color in the presence of Hg^2+^ ions, demonstrating a selective sensitivity towards this metal ion [43]. Nanocomposites, such as *A. vulgaris*-synthesized CuO-ZnO NPs, catalyze the degradation of MB more efficiently than pure ZnO NPs. Nepal et al. [40] attributed this to the heterojunction formed between the surfaces of ZnO and CuO semiconductors, which facilitates the photodegradation of dyes. *A. stelleriana*-synthesized ZnO nanoparticles demonstrated strong detoxification activity by effectively converting toxic textile dyes into harmless substances through biodegradation. This efficacy was proven by the significant restoration of *V. radiata* growth, where nanoparticle treatment increased shoot and radicle lengths by up to 87.4% and 76.4%, respectively. Additionally, nanoparticles notably reduced aquatic toxicity, resulting in significantly lower mortality rates for *A. salina* in treated solutions compared to untreated effluents [45]. Also, other biosynthesized ZnONPs degraded the reactive yellow 145 (RY-145), reactive red 120 (RR-120), RB-220 and RB-222A dyes under UV light exposure with efficiencies of 99, 95, 94 and 45%, respectively [45]. The photocatalytic behavior of *A. kopetdaghensis*-mediated AgNPs was evaluated via the degradation of MB, under UV irradiation. The process was monitored at 30 min intervals for a total of 150 min. The results indicated that AgNPs are efficient photocatalysts for removing industrial dyes. Under optimal conditions, a high degradation efficiency of 83.11% was observed [82].

## 5. Conclusions

*Artemisia* species are valuable sources for the green synthesis of metal and metal oxide nanoparticles due to their richness in bioactive constituents. The studies reviewed displayed the effectiveness of various *Artemisia* species in producing NPs (AgNPs, AuNPs, ZnONPs, Fe_2_O_3_NPs, MgONPs, MnNPs, CuONPs, SeNPs, TONPs) with applications in medicine (e.g., antimicrobial, antioxidant, and anticancer activities), agriculture, food safety, and environmental remediation. However, the current literature indicates that there are still few studies on certain *Artemisia* species and specific types of nanoparticles. Furthermore, there are limitations regarding homogeneity, size control, and the achievement of a uniform spherical shape, highlighting the need for further research to optimize synthesis parameters.

## Data Availability

The original contributions presented in this study are included in the article. Further inquiries can be directed to the corresponding author.

## Abbreviations

The following abbreviations are used in this manuscript:

AAEs	Ascorbic Acid Equivalents
ABTS	2,2′-azino-bis(3-ethylbenzothiazoline-6-sulfonic acid)
AES	Auger Electron Spectroscopy
AFM	Atomic Force Microscopy
AgNPs	Silver Nanoparticles
AuNPs	Gold Nanoparticles
CUPRAC	Cupric Reducing Antioxidant Capacity
CuNPs	Copper Nanoparticles
CuONPs	Copper Oxide Nanoparticles
DLS	Dynamic Light Scattering
DPPH	2,2-diphenyl-1-picrylhydrazyl
DW	Dry Weight
EDX/EDS	Energy-Dispersive X-ray Spectroscopy
Fe_2_O_3_NPs/Fe_3_O_4_NPs	Iron Oxide Nanoparticles
FESEM	Field Emission Scanning Electron Microscopy
FRAP	Ferric Reducing Antioxidant Power
FTIR	Fourier Transform Infrared Spectroscopy
HR-TEM	High-Resolution Transmission Electron Microscopy
IC_50_	Half Maximal Inhibitory Concentration
ICP-OES	Inductively Coupled Plasma Optical Emission Spectroscopy
IONPs	Iron Oxide Nanoparticles
IZ	Inhibition Zone
MgONPs	Magnesium Oxide Nanoparticles
MIC	Minimum Inhibitory Concentration
MnNPs	Manganese Nanoparticles
MB	Methylene Blue
MO	Methyl Orange
NCs	Nanocomposites
NiONPs	Nickel Oxide Nanoparticles
NPs	Nanoparticles
PDI	Polydispersity Index
ROS	Reactive Oxygen Species
SAED	Selected Area Electron Diffraction
SEM	Scanning Electron Microscopy
RB-220	Reactive Blue-220
RB-222A	Reactive Blue-222A
RY-145	Reactive Yellow-145
SeNPs	Selenium Nanoparticles
SPM	Scanning Probe Microscopy
SPR	Surface Plasmon Resonance
STM	Scanning Tunneling Microscopy
TAC	Total Antioxidant Capacity
TEM	Transmission Electron Microscopy
TGA	Thermogravimetric Analysis
TiO_2_NPs	Titanium Dioxide Nanoparticles
TONPs	Tin Oxide Nanoparticles
UV-Vis	Ultraviolet–Visible Spectroscopy
VSM	Vibrating Sample Magnetometer
XPS	X-ray Photoelectron Spectroscopy
XRD	X-ray Diffraction
ZCNPs	Zinc–Copper Nanoparticles
ZnONPs	Zinc Oxide Nanoparticles

## Appendix A

**Table A1:** Main classes of phytoconstituents and their bioactive compounds from the *Artemisia* genus [13,94,95,96,97,98,99,100,101].

**Monoterpenes**	
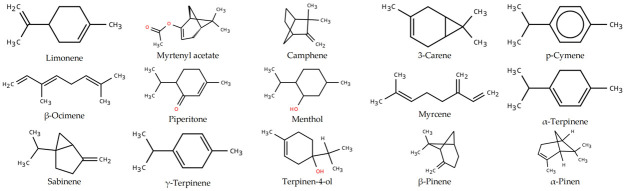
**Monoterpenoids**	
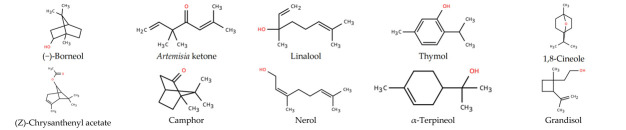
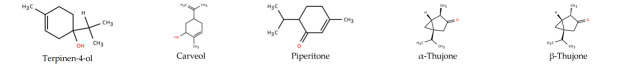
**Sesquiterpenes**	
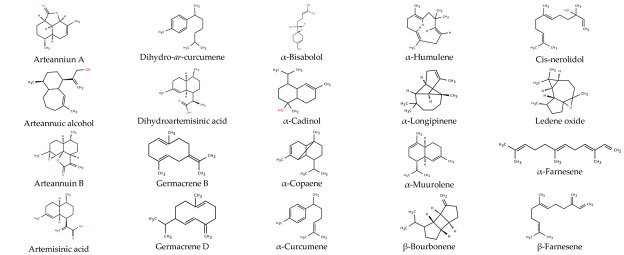
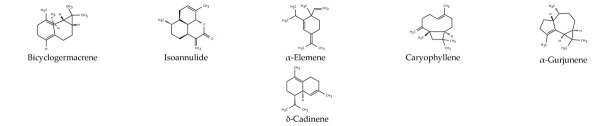
**Sesquiterpenoids**	
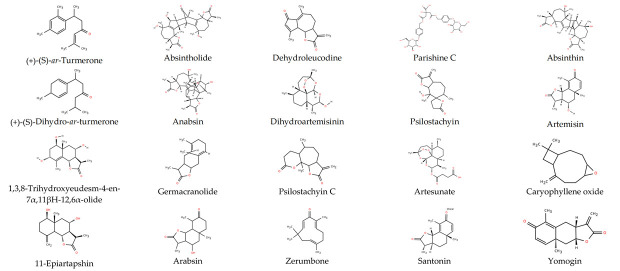
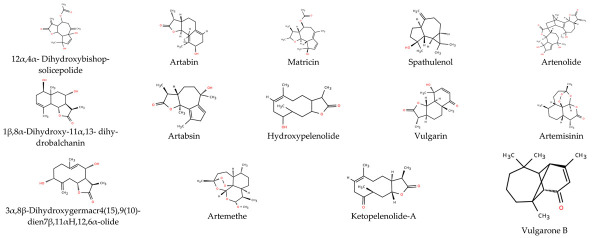
**Diterpenoids**	
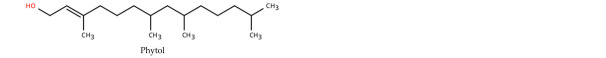
**Triterpenes**	
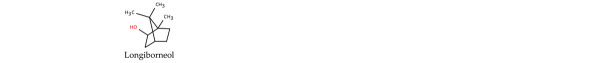
**Triterpenoids**	
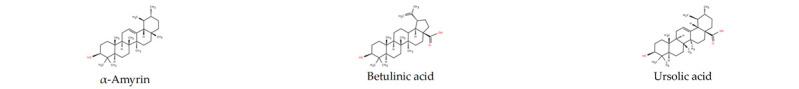
**Flavonoids**	
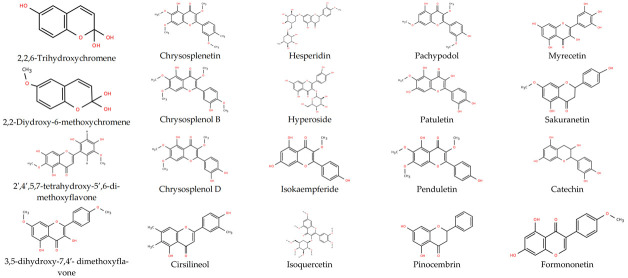
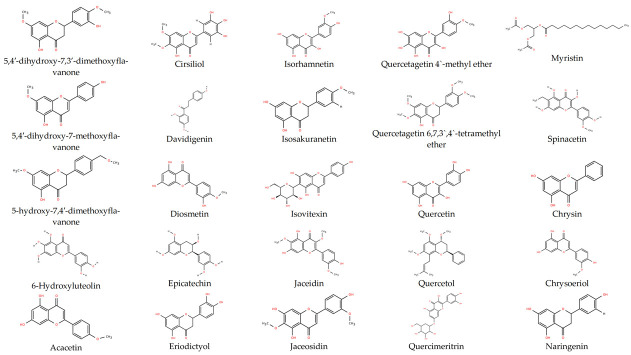
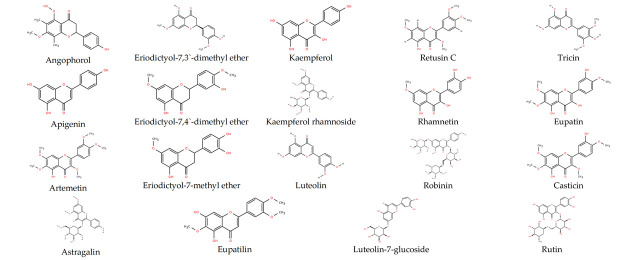
**Phenolic acids**	
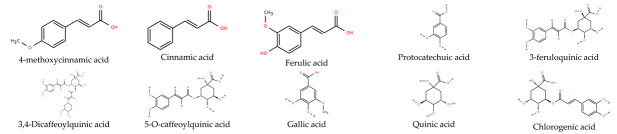
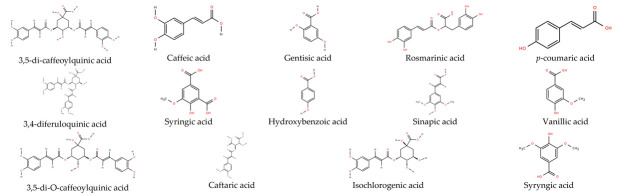
**Coumarins**	
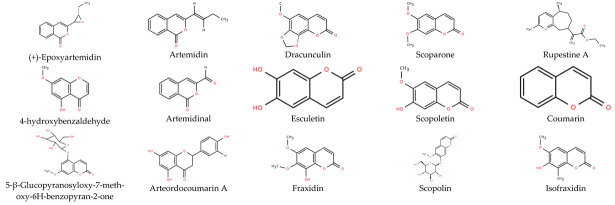
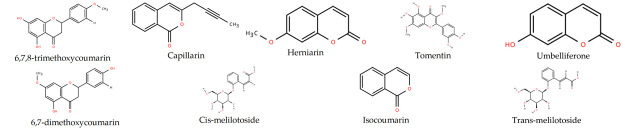
**Alkaloids**	
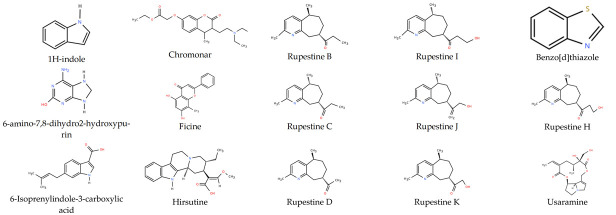
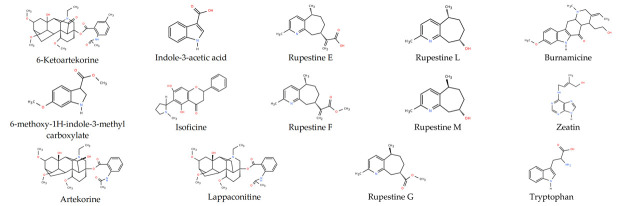
**Essential oils**	
